# Improved biodegradability of hardly-decomposable wastewaters from petrochemical industry through photo-Fenton method and determination of optimum operational conditions by response surface methodology

**DOI:** 10.1186/s13036-018-0104-9

**Published:** 2018-06-20

**Authors:** Mahmood Derakhshan, Mojtaba Fazeli

**Affiliations:** Faculty of Civil, Water and Environmental Engineering, Shahid Beheshti University A.C, Abbaspour Boulevard, Hakimieh, Tehranpars, Tehran, 17765-1719 Iran

**Keywords:** Petrochemical wastewater, Photo-Fenton, Hardly-decomposable, Biodegradation

## Abstract

**Background:**

Petrochemical wastewaters are highly polluting due to having various destructive materials such as aromatic hydrocarbons and heavy metal ions. Therefore, they need to be treated before disposal to the environment. However, due to low biodegradability, applying common treatment methods such as activated sludge is not feasible for these wastewaters.

**Methods:**

Photo-Fenton is an advanced oxidation process which was applied to promote the biodegradability of hardly-decomposable petrochemical wastewaters. The wastewater samples were provided by Maroon and Karoon petrochemical plants, located in Mahshahr, Iran. To design the experiments and analyze the experimental results, response surface method with four variables (input COD and TDS concentrations and injected a dosage of H2O2 and Fe2+) and four fixed parameters (temperature, pH, retention time, and UV power) were used.

**Results:**

The ranges of input COD, H2O2, Fe2+ and TDS were 1000 to 2500 mg L− 1, 1000 to 4000 mg L− 1, 500 to 3000 mg L− 1, and 4500 to 11,500 mg L− 1, respectively. Average input BOD5/COD ratio was 0.09. These ranges and values were determined according to the quality of the raw wastewater and experimental design. Output BOD5/COD ratio was varying between 0.3 and 0.6, which declined with an increase of input COD. The results showed that the biodegradability of the industrial wastewater was promoted upon application of higher H2O2 and Fe2+ concentrations. Meanwhile, TDS concentration had no significant effect on biodegradability of this wastewater. The following optimum conditions were resulted by evaluating the maximum efficiency of the reactor in enhancing the biodegradability of the wastewater: 1000 mg L− 1 input COD, 2668 mg L− 1 H2O2, 1655 mg L− 1 Fe2+, 8000 mg L− 1 TDS, 0.6 output BOD5/COD, 852 mg L− 1 output BOD5 and 939 mg L− 1 output COD.

**Conclusion:**

Photo-Fenton method is highly efficient for increasing the biodegradability of petrochemical wastewaters before applying biological wastewater treatment.

## Background

Due to the existence of hazardous pollutants such as aromatic hydrocarbons in petrochemical wastewaters, direct discharge of them to the environment can contaminate the resources and threaten the health of species that live in their vicinity [1]. Hence, discharging petrochemical wastewaters with hazardous pollutants such as petrochemicals and hydrocarbons should follow specific rules and standards [2]. In this respect, petrochemical complexes are under pressure by environment protection campaigns and organizations to reduce their pollutants emission, reuse water, and decrease power consumption to decline carbon effects [3]. In this regard, many studies have attempted to develop new ecofriendly treatment methods with maximum efficiency of pollutant removal, separation, and disposal.

Using microalgal systems for nutrient removal [4, 5], aerobic granular sludge [6], using photocatalytic reactors [7] and applying anoxic/oxic process [8, 9] are the newest efforts which are carried out in order to treat petrochemical wastewaters efficiently. As it is obvious, due to the level of organic load and the variety of the contaminants in the wastewater streams in petrochemical industry, biological treatment techniques seem to be inevitable [10]. However, due to existence of different non-biodegradable compounds (mainly hydrocarbons) in petrochemical wastewaters, common biological treatment methods (e.g. activated sludge) are not practically feasible for being applied as the only method of treatment and an efficient pretreatment method is usually necessary to improve the biodegradability of wastewater [11]. Therefore, accelerating techniques which significantly improves wastewater biodegradability are usually employed to solve the problem of wastewater treatment in petrochemical complexes [12].

Advanced oxidation processes (AOPs) can be considered as an effective treatment method to deal with hardly-biodegradable pollutants in wastewaters from the petrochemical industry [13]. Such processes have recently received much attention because of their capabilities of pollutant removal and converting hardly-decomposable materials to biodegradable materials [14]. These methods are based on producing radicals that are highly reactive, non-selective and with high oxidation potency [15]. In general, these methods use free radicals as strong oxidants to break long chain and hardly-decomposable organic molecules to smaller ones, which often offer improved biodegradability and can be used to generate carbon dioxide upon complete degradation [16]. In AOPs, oxidizing agents such as ultrasound, UV, O_3_, TiO_2_, and H_2_O_2_ are utilized solely or in combination with each other [17]. These methods are often combined with or complement to each other and are used prior to or after other major treatment processes [18]. It has to be noted that when using chemical oxidants, a complete oxidation of the pollutants is not concerned due to the related high costs. Hence, partial oxidation is an effective alternative to enhance biological treatment of specific compounds [19].

There are numerous oxidation methods for decomposition of hardly-biodegradable pollutants in complex wastewater such as petrochemical wastewaters [20]. Photo-Fenton is an effective method which is noticeably efficient for chemical oxidation of hardy-decomposable compounds in wastewater [21, 22]. Classical Fenton reaction is a combination of hydrogen peroxide (H_2_O_2_) and Ferro ions (Fe^2+^) in an acidic medium that results in cleavage of H_2_O_2_ into hydroxyl ion and hydroxyl radical and oxidation of Fe^2+^ to Fe^3+^ [23, 24]. The reaction takes place at room temperature without input energy [25]. Also, this reaction requires a relatively short time compared to other AOPs [26]. Besides, the needed reactants are readily accessible, can be conveniently stored, and are safe for maintenance [27]. Fenton reaction processes are prompted upon light irradiation and changed to a process known as photo-Fenton [28, 29].

In the presence of UV light, a greater number of OH radicals are generated from both direct photolysis of H_2_O_2_ and UV radiative reaction with iron, in aqueous solutions [30] (Eq. 1–3). Therefore, in photo-Fenton process, more OH radicals can be produced and efficiency enhances consequently [31]. This method is based on OH radical generation, which acts as a very strong oxidant in the destruction of compounds that cannot be oxidized with common oxidants such as O_2_ and O_3_ [32]. These radicals react with the compounds in solution and initiate a set of oxidation reactions until the compounds reach to the desired decomposition degree or convert to inorganic compounds, completely [33]. The radical has a high oxidation tendency toward most materials, without resistance to any material or a specific group.

1 $$ {\mathrm{H}}_2{\mathrm{O}}_2+\mathrm{UV}\to 2\ \mathrm{OH} $$

2 $$ {\mathrm{Fe}}^{3+}+{\mathrm{H}}_2\mathrm{O}+\mathrm{UV}\to {\mathrm{Fe}}^{2+}+{\mathrm{H}}^{+}+{\mathrm{OH}}^0 $$

3 $$ \mathrm{Fe}{{\left(\mathrm{OH}\right)}_2}^{+}+\mathrm{UV}\to {\mathrm{Fe}}^{2+}+{\mathrm{OH}}^0 $$

Furthermore, OH radicals have gained attraction because of their oxidation power, reasonable operational cost, water solubility, and avoiding production of toxic side products. Convenience, simplicity, and efficiency of Fenton process at constant removal rate have been proved elsewhere [34].

Different Fenton-based oxidation processes in petrochemical wastewater treatment have drawn more attention to themselves during last years. Davarnejad et al. (2014) had applied electro-Fenton method using aluminum and iron electrodes in order to treat petrochemical wastewater and had achieved 67 and 54% of COD removal for iron and aluminum respectively [35]. In another study, Wang et al. (2015) had applied Fenton method in order to increase the biodegradability of petrochemical wastewater and had reduced the number of the polluting compounds in the wastewater from 117 to 27 most of which were biodegradable components [36]. Scaratti et al. (2017) applied heterogeneous photo-Fenton process for synthetic petrochemical wastewater using residue-based iron oxide nanocatalysts and graphene oxide in order to compare the influences of these two nanostructures on TOC and COD removal [37].

In this research, it is tried to increase the biodegradability of petrochemical wastewater using photo-Fenton process. The raw wastewaters from petrochemical plants of Maroon and Kroon in Mahshar petrochemical and economic zone, Iran, were used. Since BOD_5_/COD ratios of these wastewaters are low, they are categorized as hardly-biodegradable wastewaters and common biological treatment methods are not feasible for their treatment. The objective of this research is to enhance the biodegradability of petrochemical wastewaters through enhancing BOD_5_/COD ratio by photo-Fenton method. Applying photo-Fenton method in order to increase the biodegradability as a pre-treatment method for actual petrochemical wastewater is the main objective of this research.

## Methods

### Experimental setup

**Fig. 1:**
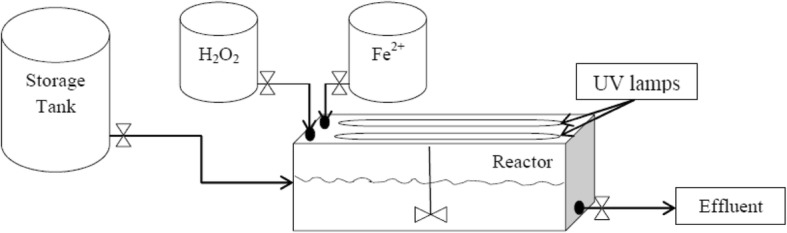
Schematic illustration of the photo-Fenton reactor

In this study, a rectangular cube glassy reactor with 80 cm length, 20 cm width and 20 cm height was used (Fig. 1).

A stirrer was placed on the top of the reactor for a complete mixing of the wastewater with chemicals. Moreover, UVC lamps with 60 cm length and 20 W powers (Philips, Netherlands) were installed in the center of the reactor. Inlets of hydrogen peroxide and Fe^2+^ are shown in Fig. 1. The experiments were considered to be in a batch state with 1 h retention time.

Fluid level height was 5 cm and the distance between the UV lamp and the fluid surface was 15 cm. According to the height and length of the fluid (5 and 60 cm, respectively), the volume of the reactor was considered to be 6000 cm^3^. Based on literature review and field studies, the retention time of 1 h, the temperature of 25 ± 2 °C, and pH = 3 were concerned as fixed parameters.

### Chemicals and the specification tests

The chemicals used in this study included hydrogen peroxide (Merck, 30% *w*/w and MW = 34 g mol^− 1^), hydrated iron sulfate (FeSO_4_.7H_2_O), methanol (CH_3_OH), sodium hydroxide (NaOH), and sulfuric acid (H_2_SO_4_). COD and BOD_5_ were measured at the reactor inlets and outlets. Also, TDS, H_2_O_2_, Fe^2+^, pH, and temperature were measured based on standard methods [38].

### Wastewater composition

Specification of the utilized raw wastewater are listed in Table 1. This wastewater was sampled from a mixture of output wastewaters from Maroon and Karoon petrochemical plants with 2 to 1 ratio. Sampling was performed in spring of 2016. To explore parameter properties in other seasons, when lower pollution level is usually expected, experimental trials of treatment system were used. The most remarkable feature of the used wastewater was a considerable amount of hardly-decomposable materials and a high TDS level.

**Table 1:** Properties of the natural wastewater used in the experiments

Parameter	Industrial wastewater		Sanitary wastewater	Mix wastewater for experimental
	Karoon petrochemical	Maroon petrochemical	Karoon petrochemical	
COD (mg L^−1^)	3980	742	81	2498
BOD_5_ (mg L^−1^)	345	70	64	226
TDS (mg L^−1^)	7050	24,350	1402	11,186
pH	5	6.5	7	5.7
BOD_5_/COD	0.09	0.09	0.8	0.09
Mix ratio	2	1	0.5	–

As shown in Table 1, the sampled wastewaters were employed with the outlined ratios to achieve qualitative characteristics required for mixture experiments. According to Eq.4, different parameters with experimental responses were compared to acquire mixed raw wastewater.

4 $$ \left({P}_{im}\times 2\right)+\left({P}_{ik}\times 1\right)+\left({P}_{sk}\times 0.5\right)=\left({P}_m\times 3.5\right) $$

where P_im_ is the parameter regarding Maroon petrochemical industry wastewater, P_ik_ is the intended parameter corresponding to Karoon petrochemical industry wastewater, P_sk_ is the parameter related to Karoon sanitary petrochemical wastewater, and P_m_ is the parameter of interest associated with the mixed wastewater.

Based on the documents provided by the laboratory of the purgation unit for the other seasons of the year and also Table 1, the employed ranges of COD and TDS were selected to be 1000 to 2500 mg L^− 1^ and 4500 to 11,500 mg L^− 1^, respectively.

### Experiments conditions and procedure

Experiments were conducted at common lab temperature of 25 ± 2 °C. The pH of all the samples which are entering the reactor were adjusted on 3 using 0.1 M NaOH and 0.1 M H_2_SO_4_ [39]. Then, a specific amount of hydrated iron sulfate was added to the reactor. The sample was then mixed with the chemicals and homogenized with the electrical stirrer. The materials were dissolved in the wastewater under UV radiation for 30 min. Photo-Fenton experiment was initiated by gradual addition of hydrogen peroxide (H_2_O_2_) [29]. To prevent excess reactions in the sample, 0.1 mL methanol was added to the reactor, at the end of the experiment [40]. Eventually, COD and BOD_5_ of the outlets were measured by HACH DR/2800 and HACH BOD (HACH, US) instruments, respectively. The procedure was repeated for all trials.

### Experimental design and statistical analysis

Response surface method (RSM) has been frequently used to perform statistical analysis and define optimum conditions. The advantages of this method are a reduced number of necessary experiments for result analysis and decreased experimental attempts [41]. In this study, central composite design (CCD) approach was adopted to carry out multi-variable modeling and analysis [42]. The experiment design in this study was through CCD method and by relying on four variable parameters of inlet COD concentration, H_2_O_2_ concentration, Fe^2+^ injected dosage and TDS concentration. Also, Design Expert® 10.01 software was used to devise the experiments, carry out statistical analysis, model and determine optimum conditions. The variables were analyzed at 5 levels (Table 2). The suggested number of experiments was randomly obtained to be 54 trials by considering 6 central repetitions and 2 repetitions in other points. Interactions between the variables and variance analysis of the data (ANOVA) were performed through RSM.

**Table 2:** The levels used in the parameters used in the test

Parameters	-alpha	Low	Center	High	+alpha
COD initial	1000	1375	1750	2125	2500
H_2_O_2_	1000	1750	2500	3250	4000
Fe^2+^	500	1125	1750	2375	3000
TDS	4500	6250	8000	9750	11,500
(BOD/COD)_IN_	0.09				
pH	3				

Quality of the model and the predicted values can be determined by an R^2^ coefficient. The variables used in this part are recorded by Eq.5.

5 $$ {X}_i=\frac{\left({x}_i-{x}_{cp}\right)}{\Delta  {x}_i} $$

where X_i_ is the encoded value of the i-th independent parameter, x_i_ the desired independent parameter, x_cp_ is the value of the independent parameter in the central point, and *∆*x_i_ is the value of the parameter change in each step. In the RSM method, the general form of the quadratic fitted equation is in accordance with Eq.6 [43].

6 $$ Y={\beta}_0+\sum \limits_{i=1}^k{\beta}_i{X}_i+\sum \limits_{i=1}^k{\beta}_{ii}{X}_i^2+\sum \limits_{i<j}^k{\beta}_{ij}{X}_i{X}_j+\varepsilon $$

where *Y* is the response variable and BOD_5_/COD ratio in the reactor outlet, *β*_*0*_ is a constant, *β*_*i*_ is linear coefficient, *β*_*ii*_ refers to the second order effect of the model, *β*_*ij*_ corresponds to the coefficient of mutual interaction and _ε_ is the statistical error.

## Results and discussion

### Development and regression equations of the model

The basis of the experimental procedure is the varying values of the independent variable, which was defined according to experimental results and literature review. Experimental design and the measured response are reported in Table 3. As the results illustrate, the empirical relationship between the independent variables and the response was obtained to be Eq.7 using Design expert software, in the framework of CCD method.

7 $$ \frac{BOD_5}{COD}=0.43-0.059\times A+0.046\times B+0.030\times C+\left(5.829E-003\right)\times BC+0.014\times {A}^2-0.012\times {B}^2-0.010\times {C}^2 $$

**Table 3:** Experiment design according to RSM method and the measured response

Test number	Input variable parameters (mg L^−1^)				Response	Test number	Input variable parameters (mg L^− 1^)				Response
	COD	H_2_O_2_	Fe^2+^	TDS	BOD/COD		COD	H_2_O_2_	Fe^2+^	TDS	BOD/COD
1	1750	1000	1750	8000	0.299756	28	1750	4000	1750	8000	0.451106
2	1375	1750	1125	9750	0.385659	29	2500	2500	1750	8000	0.356573
3	2125	3250	1125	9750	0.374284	30	1750	2500	1750	8000	0.474479
4	1750	2500	1750	11,500	0.421166	31	1750	2500	1750	8000	0.396539
5	1375	1750	1125	9750	0.38932	32	1375	3250	1125	6250	0.503843
6	1375	3250	2375	6250	0.581491	33	1375	3250	1125	6250	0.501281
7	1750	4000	1750	8000	0.44911	34	1375	1750	1125	6250	0.388727
8	1750	2500	1750	4500	0.424155	35	2125	3250	1125	6250	0.374841
9	1750	2500	1750	8000	0.37437	36	2125	1750	2375	6250	0.336815
10	1375	3250	2375	9750	0.581357	37	1375	1750	2375	9750	0.444746
11	1000	2500	1750	8000	0.59867	38	2125	3250	2375	6250	0.428827
12	2125	1750	1125	9750	0.300457	39	2125	3250	1125	6250	0.374444
13	1750	2500	1750	8000	0.460101	40	1375	1750	2375	9750	0.444746
`14	1375	3250	2375	9750	0.581357	41	2125	3250	2375	9750	0.426278
15	1375	1750	2375	6250	0.444545	42	1375	1750	2375	6250	0.443841
16	2125	1750	1125	6250	0.301307	43	1750	2500	1750	11,500	0.422882
17	1750	2500	500	8000	0.319115	44	2125	1750	2375	9750	0.337247
18	1000	2500	1750	8000	0.599777	45	2500	2500	1750	8000	0.356953
19	1750	2500	3000	8000	0.441197	46	1750	2500	3000	8000	0.442775
20	1375	3250	2375	6250	0.581833	47	2125	1750	2375	9750	0.337247
21	2125	3250	2375	6250	0.426786	48	2125	1750	1125	9750	0.30085
22	2125	3250	2375	9750	0.426357	49	2125	1750	2375	6250	0.337467
23	2125	1750	1125	6250	0.30085	50	1750	2500	500	8000	0.319653
24	1375	3250	1125	9750	0.470356	51	2125	3250	1125	9750	0.375318
25	1750	2500	1750	8000	0.422495	52	1375	1750	1125	6250	0.387379
26	1750	2500	1750	8000	0.422799	53	1750	1000	1750	8000	0.300979
27	1750	2500	1750	4500	0.422799	54	1375	3250	1125	9750	0.501288

Regression analysis was performed on the appropriate performance of the response. In the developed second order equation, A, B, and C stand for COD, H_2_O_2_, and Fe^2+^, respectively. Eq.7 describes how BOD_5_/COD ratio is affected by each individual input variable or by mutual interaction of the variables. BOD_5_/COD ratio demonstrates linear and second order variations with respect to COD, H_2_O_2_, and Fe^2+^, suggesting that the input independent parameters interact with each other [44].

**Fig. 2:**
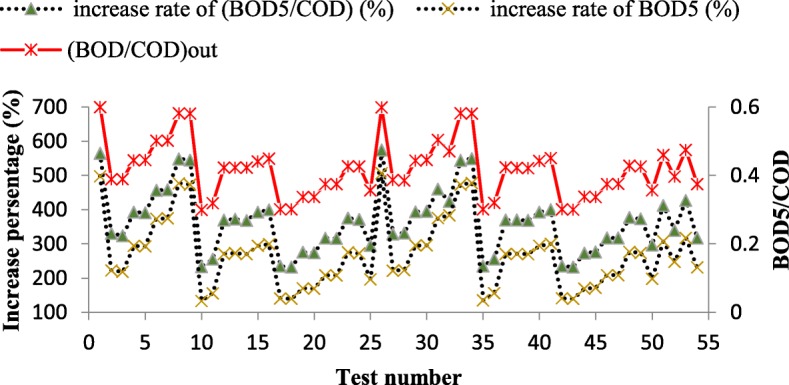
The Photo-Fenton method results in increase BOD5 and (BOD5/COD) ratio

As shown in Fig. 2, the photo-Fenton method has been very successful in the promoting the biodegradability of petrochemical wastewater. The rate of increase in all tested samples was more than 100%, which in the most rate increased as 506%. The BOD_5_/COD ratio also increased from 232 to 574%.

### Analysis of variance of the data (ANOVA)

ANOVA results (Table 4) show the success of the model and influence of the input factors on each other and the employed response.

**Table 4:** Analysis of variance (ANOVA) for BOD_5_/COD

Source	SUM of Square	Degree of Freedom	Mean Square	F Value	P- Value *Prob* > *F*	
Model	0.34	7	0.049	151.26	<0.0001	significant
A-COD	0.17	1	0.17	520.54	<0.0001	
B-H_2_O_2_	0.10	1	0.10	319.78	<0.0001	
C-Fe^2+^	0.042	1	0.042	130.14	<0.0001	
BC	1.087E-003	1	1.087E-003	3.36	0.0732	
A^2^	9.264E-003	1	9.264E-003	28.65	<0.0001	
B^2^	6.679E-003	1	6.679E-003	20.66	<0.0001	
C^2^	5.226E-003	1	5.226E-003	16.16	0.0002	
Residual	0.015	46	3.233E-004			
Lack of Fit	7.308E-003	17	4.299E-004	1.65	0.1149	not significant
Pure Error	7.565E-003	29	2.608E-004			
Cor Total	0.36	53				
R^2^ = 95.84%						
$$ {R}_{adj}^2=95.20\% $$						

The accuracy of the results and their consistency with the model was investigated through F values of the model and Lack of Fit. A model is accurate when its P and F values are significant while its Lack of Fit is insignificant [45]. F value of the model is 151.26 which is significant. There is 0.01% probability of F value error. Prob > F values below 0.0500 show that the model parameters are statistically significant. In this study, the important A, B, C, A^2^, B^2^, and C^2^ parameters are reported. F value for Lack of Fit is reported to be 1.65 that is negligible compared to the net error. There is an 11.49% possibility that a part of F value in Lack of Fit result from the error. The insignificance of Lack of Fit is a good sign and declares the validity of the model. It means that the model relationships are statistically significant and can predict satisfactory responses.

Model consistency was evaluated by determining R^2^ coefficient. In this study, R^2^ was obtained to be 0.9584 for BOD_5_/COD ratio. As R^2^ approximates unity, the model is stronger and the predicted responses are more reliable. The R^2^_adj_ value was calculated to be 0.9520 that is a high value for biological decomposition and indicates significance and appropriateness of the model. R^2^_pred_ was 0.9454 that differs from the R^2^_adj_ value as much as 0.2. This difference is logically acceptable. The low value of model’s variance coefficient (C.V%), i.e. 4.3, demonstrates precision and proper validity of the experiments [46, 47].

### Interactive impacts between the variables

**Fig. 3:**
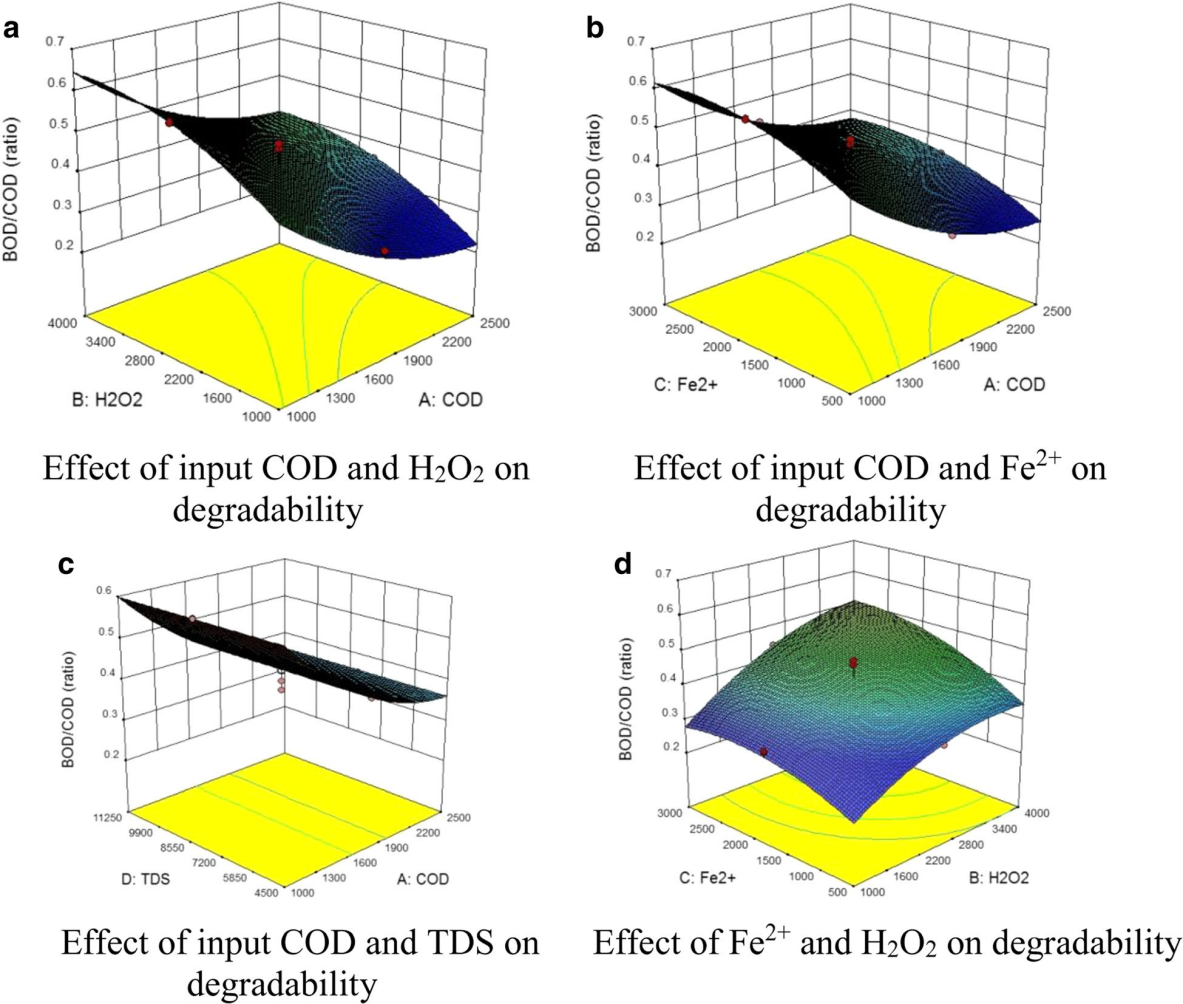
Effect of the variable parameters on degradability of wastewater in the outlet of photo-Fenton process. **a** Effect of input COD and H_2_O_2_ on degradability. **b** Effect of input COD and Fe^2+^ on degradability. **c** Effect of input COD and TDS on degradability. **d** Effect of Fe^2+^ and H_2_O_2_ on degradability

Influence of effective variable parameters on photo-Fenton process (i.e., COD, H_2_O_2_, Fe^2+^, and TDS) on degradation extent of hardly-decomposable organic compounds to molecules with high degradability is explained as follows. Fig. 3 presents interaction impacts of the selected parameters on degradability as a 3D graph.

Based on Fig. 3, input COD, H_2_O_2_, and Fe^2+^ parameters pose a noticeable impact on degradability but TDS does not show an effective role in biodegradation of wastewater contents. According to the results, the highest influence is due to input COD, Fe^2+^, and H_2_O_2_, respectively.

### Evaluation of the effect of each independent variable on degradability

**Fig. 4:**
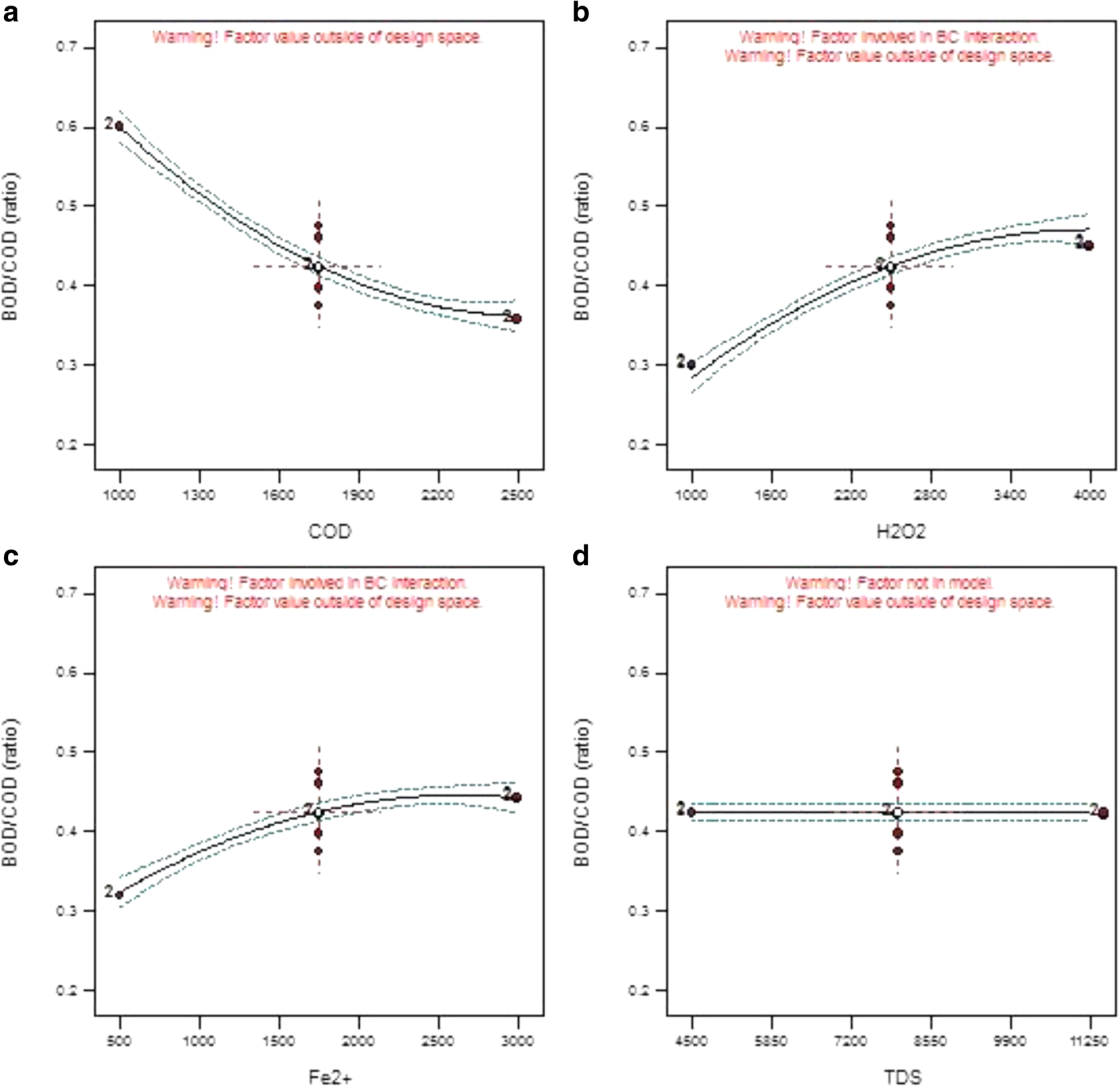
Effect of each independent parameter on output BOD5/COD. **a** COD. **b** H202. **c** Fe2+. **d** TDS

The effect of each parameter on output BOD_5_/COD is depicted in Fig. 4. The experiments on each parameter were performed at 5 different levels. As shown in Fig. 4a, wastewater degradability has decreased with input COD increase. This phenomenon is a consequence of the increased amount of materials that can be hardly decomposed in the wastewater inlet with an elevated amount of input COD. As less hardly-decomposable materials are in the wastewater, the OH radicals generated during photo-Fenton process decompose them more feasibly and widely. Accordingly, at the defined time, the organic molecules will be converted into biodegradable molecules more noticeably. The reported BOD_5_/COD ratio of this section varied from 0.36 to 0.60.

As can be seen from Fig. 4b, output BOD_5_/COD ratio increases with H_2_O_2_ concentration up to 3250 mg L^− 1^. This trend is an outcome of a promoted OH radical generation with accessibility to more H_2_O_2_ molecules. As can be seen from the figure, a degradability of 0.30 to 0.45 was achieved. According to Fig. 4c, higher concentrations of Fe^2+^ give an ascending trend of degradability due to a higher rate of OH radical production. The BOD_5_/COD ratio was reported to be within the range of 0.32 to 0.44. No BOD_5_/COD increasing or decreasing effect was observed for TDS parameter on photo-Fenton process. In all conducted evaluations, the BOD_5_/COD ratio of the wastewater was reported to be 0.3 to 0.6 in photo-Fenton process. This finding demonstrates a high efficiency of photo-Fenton process in the provision of biodegradability for wastewaters with high contents of hardly-decomposable organic materials in saline mediums.

### Optimum conditions

According to the obtained results, selection of the optimum values of each parameter is crucial for the efficient performance of the reactor in purgation systems. Therefore, the outlined model was used to identify the optimum status in order to reach high degradability of the wastewater, from both operational and economic perspectives. According to Table 5, the values of H_2_O_2_, and Fe^2+^ consumption, which are economically important, were adjusted to their minimum values. Moreover, input COD and TDS, which varied with respect to the input wastewater, were kept in a certain range and the BOD_5_/COD ratio was considered to be the highest possible value in selecting the optimum points. A total of 24 optimum points were achieved by the model, among which the state provided in Table 6 was the best point.

**Table 5:** Selection of different economic and operational statuses for an optimum point

Parameter	GOAL	Importance
Inlet COD	in range	***
H_2_O_2_	minimize	***
Fe^2+^	minimize	***
TDS	in range	***
BOD_5_ (OUT)	in range	***
COD (OUT)	in range	***
BOD_5_/COD (ratio)	maximize	*****

**Table 6:** The most appropriate observed optimum condition for promotion of biodegradability

Inlet COD (mg L^−1^)	H_2_O_2_ (mg L^−1^)	Fe^2+^ (mg L^−1^)	TDS (mg L^−1^)	Outlet COD (mg L^−1^)	Outlet BOD_5_ (mg L^−1^)	BOD_5_/COD (ratio)
1000.04	2668.09	1655.44	5287.44	939	582	0.6

As can be observed in Table 6, wastewater degradability is quite successful under optimum conditions. By maintaining the conditions of the wastewater entering treatment unit at the suggested values, the photo-Fenton process will provide a biological wastewater that can be treated by biological methods efficiently.

## Conclusion

Using a successful modeling by Design Expert 10.0.1, it was found that three parameters of input COD, H_2_O_2_, and Fe^2+^ have great impacts on conversion of hardly-decomposable materials into biodegradable molecules that can be purged by biological methods. TDS that is an indication of medium salinity did not pose any significant effect on photo-Fenton process. In all stages of the experiments by photo-Fenton method, BOD_5_/COD ratio varied from 0.3 to 0.6. These values demonstrate the suitability of photo-Fenton process for decomposition of petrochemical industry wastewater into biodegradable materials in any experimental condition. The BOD_5_/COD ratio was reduced with input COD increase but enhanced by increasing injection dosages of H_2_O_2_ and Fe^2+^. The maximum BOD_5_/COD ratio was reported to be 0.6 that the high potential of photo-Fenton process in the decomposition of the wastewater materials into biodegradable compounds.

## Abbreviations

ANOVA: Analysis of variance
AOP: Advanced oxidation process
BOD: Biological oxygen demand
CCD: Central composite design
COD: Chemical oxygen demand
RSM: Response surface methodology
TDS: Total dissolved solids
UV: Ultra violet
